# Responses of *Ottelia alismoides*, an aquatic plant with three CCMs, to variable CO_2_ and light

**DOI:** 10.1093/jxb/erx064

**Published:** 2017-03-21

**Authors:** Hui Shao, Brigitte Gontero, Stephen C Maberly, Hong Sheng Jiang, Yu Cao, Wei Li, Wen Min Huang

**Affiliations:** 1 Key Laboratory of Aquatic Botany and Watershed Ecology, Wuhan Botanical Garden, Chinese Academy of Sciences, Wuhan, China; 2 Aix Marseille Univ CNRS, BIP UMR, IMM, FR, Chemin Joseph Aiguier, Marseille Cedex, France; 3 Lake Ecosystems Group, Centre for Ecology & Hydrology, Lancaster Environment Centre, Library Avenue, Bailrigg, Lancaster, UK; 4 Hubei Key Laboratory of Wetland Evolution & Ecological Restoration, Wuhan Botanical Garden, Chinese Academy of Sciences, Wuhan, China

**Keywords:** Aquatic plant, C_4_, CAM, CCMs, PEPC, photosynthesis, PPDK, Rubisco, starch

## Abstract

*Ottelia alismoides* is a constitutive C_4_ plant and bicarbonate user, and has facultative crassulacean acid metabolism (CAM) at low CO_2_. Acclimation to a factorial combination of light and CO_2_ showed that the ratio of phosphoenolpyruvate carboxylase (PEPC) to ribulose-bisphosphate carboxylase/oxygenase (Rubisco) (>5) is in the range of that of C_4_ plants. This and short-term response experiments showed that the activity of PEPC and pyruvate phosphate dikinase (PPDK) was high even at the end of the night, consistent with night-time acid accumulation and daytime carbon fixation. The diel acidity change was maximal at high light and low CO_2_ at 17–25 µequiv g^−1^ FW. Decarboxylation proceeded at ~2–3 µequiv g^−1^ FW h^−1^, starting at the beginning of the photoperiod, but did not occur at high CO_2_; the rate was greater at high, compared with low light. There was an inverse relationship between starch formation and acidity loss. Acidity changes account for up to 21% of starch production and stimulate early morning photosynthesis, but night-time accumulation of acid traps <6% of respiratory carbon release. *Ottelia alismoides* is the only known species to operate CAM and C_4_ in the same tissue, and one of only two known aquatic species to operate CAM and bicarbonate use.

## Teaser

*Ottelia alismoides* grown at low CO_2_ operates CAM during the night and C_4_ metabolism during the day. Night-time accumulation of acid is controlled by CO_2_. Decarboxylation is controlled by light.

## Introduction

In terrestrial environments, some photoautotrophic plants have evolved carbon dioxide-concentrating mechanisms (CCMs), such as C_4_ and crassulacean acid metabolism (CAM), that allow them to maximize carbon uptake when temperature is high or water restricted, or both (Keeley and Rundel, 2003; Herrera, 2009; Silvera *et al.*, 2010; Sage *et al.*, 2012). In contrast, freshwater plants have CCMs that overcome the problem of limited inorganic carbon supply which arises from several factors (Madsen and Maberly, 1991; Vadstrup and Madsen, 1995; Maberly and Madsen, 2002). First, the rate of CO_2_ diffusion in water is ~10 000 times lower than in air, limiting the rate of transport of CO_2_ into freshwater plants through the external boundary layer (Raven, 1970; Black *et al.*, 1981; Maberly, 2014). Consequently, the CO_2_ concentration required to half-saturate the net photosynthesis of freshwater plants is ~8–14 times greater than air equilibrium (Maberly and Madsen, 1998). Secondly, in productive systems, the concentration of CO_2_ can be depleted close to zero when the demand for inorganic carbon by community photosynthesis exceeds the supply from the atmosphere, the catchment, and heterotrophic regions such as sediments.

In addition, freshwater plants have evolved a diversity of strategies that counter CO_2_ limitation (Klavsen *et al.*, 2011). Physiological or biochemical strategies involve CCMs because they increase the concentration of CO_2_ around the active site of ribulose-bisphosphate carboxylase/oxygenase (Rubisco) (Bowes and Salvucci, 1989; Maberly and Madsen, 2002; Raven *et al.*, 2008). The most frequent CCM in freshwater plants is based on the biophysical uptake of bicarbonate, which is found in >50% of tested species (Maberly and Madsen, 2002). In addition, the two biochemical CCMs in terrestrial plants, C_4_ and CAM, are also found in freshwater plants. Both depend on carbon fixation via the enzyme phosphoenolpyruvate carboxylase (PEPC) that is active during either the day (C_4_) or the night (CAM) (Keeley, 1981; Bowes and Salvucci, 1989; Bowes *et al.*, 2002; Keeley and Rundel, 2003).

The C_4_ pathway in freshwater plants is analogous to that in terrestrial C_4_ plants, and the percentage of species with this syndrome in both systems is ~3% (Zhang *et al.*, 2014). In freshwater plants, C_4_ metabolism has been observed in *Hydrilla verticillata*, *Egeria densa*, and *Ottelia alismoides* from the Hydrocharitaceae, and in some other species including *Sagittaria subulata* from the Alismataceae, the grasses *Orcuttia californica* and *O. viscida*, and the sedge *Eleocharis acicularis* (Bowes and Salvucci, 1989; Madsen and Sand-Jensen, 1991; Keeley, 1998*a*; Casati *et al.*, 2000; Zhang *et al.*, 2014). In most terrestrial C_4_ plants, PEPC and Rubisco are located in different cells, but some species from the Chenopodiaceae, in saline semi-deserts, operate single-cell C_4_ photosynthesis (Voznesenskaya *et al.*, 2001). In freshwater plants, C_4_ occurs within a single cell in the few plants studied (Bowes, 2011). Terrestrial C_4_ is generally constitutive, while it is facultative in *H. verticillata* and *E. densa*, being induced when inorganic carbon is limiting (Van *et al.*, 1976; Reiskind *et al.*, 1997;Casati *et al.*, 2000). In contrast, it is constitutive in *O. alismoides* (Zhang *et al.*, 2014).

The CAM pathway enables plants to fix CO_2_ at night via PEPC and store it as malic acid in the vacuole. During the day, malic acid is decarboxylated and the released CO_2_ is fixed by Rubisco, entering the Calvin–Benson cycle (Cushman and Bohnert, 1999; Nimmo, 2000). The frequency of species with CAM in terrestrial and freshwater environments is ~6% (Zhang *et al.*, 2014). Freshwater CAM plants have been observed in the following genera: *Crassula*, *Deinostema*, *Isoetes*, *Littorella*, *Ottelia*, *Sagittaria*, and *Vallisneria* (Keeley, 1981, 1982, 1998*b*; Aulio, 1986; Klavsen *et al.*, 2011; Pedersen *et al.*, 2011; Zhang *et al.*, 2014; Yang and Liu, 2015; Yin *et al.*, 2017). Unlike terrestrial CAM plants where stomatal closure suppresses CO_2_ uptake during the day, daytime uptake of exogenous CO_2_ can occur in freshwater plants, allowing them to assimilate exogenous CO_2_ continuously (Keeley, 1998*b*; Madsen *et al.*, 2002). The expanded time scale for inorganic carbon uptake enhances the daily carbon supply and facilitates the recapture of respired CO_2_ at night.

CAM metabolism is a plastic process in freshwater plants (Bowes and Salvucci, 1989). Regulation can involve long-term acclimation (over weeks or months) or short-term exposure (over a 24 h cycle) to environmental change, including variation in water, light, CO_2_, temperature, and nutrients (Keeley *et al.*, 1983; Aulio, 1985; Madsen, 1987; Robe and Griffiths, 1990; Hostrup and Wiegleb, 1991; Klavsen and Maberly, 2010). CAM activity is dependent on the interactions among these environmental variables and particularly light (Robe and Griffiths, 1990; Rattray *et al.*, 1992) and CO_2_ (Richardson *et al.*, 1984; Zhang *et al.*, 2014).

*Ottelia alismoides* is a member of the Hydrocharitaceae, and, perhaps uniquely, has three CCMs: use of bicarbonate and C_4_ metabolism that are constitutive, and CAM that is facultative (Zhang *et al.*, 2014). This raises the question of how these three CCMs interact and are regulated over different time scales, especially in response to variable CO_2_ and light. The aims of this study were: (i) to investigate the long-term regulation of inorganic carbon uptake in *O. alismoides*, including the interactive effects on C_4_ and CAM, in response to variable light and CO_2_ concentration; and (ii) to assess the short-term effect of changes in light and CO_2_ on the daily CAM cycle.

## Materials and methods

### Plant material

*Ottelia alismoides* that had been collected from Yunnan Province, China, was cultivated in a greenhouse at the Wuhan Botanical Garden for several years. Seeds from these plants were sown in six plastic containers (10 × 10 × 10 cm) filled with 5 cm of soil from nearby Donghu Lake and covered with 3 cm of sterile tap water. The containers were placed in a growth chamber at 25 °C and the water was replaced completely each day to maintain constant conditions. After several days, the seeds began to germinate and, after 6 weeks, when the seedlings were ~8 cm tall, two or three plants were transplanted into a plant pot (15 cm diameter, 12 cm high) containing the same soil. Nineteen pots were placed in a tank containing ~400 litres of tap water located in a glasshouse on the flat roof of the laboratory. The tap water was replaced every 2 d and the water surface gradually increased as the plants grew. Snails and moribund leaves were removed daily. The plants were grown at ambient (high) light without shading and, because of their high biomass, they generated high pH values and low concentrations of CO_2_. After ~4–5 weeks, plants of similar height (~25–30 cm) were selected for the experiments, in July and August 2015.

### Acclimation to light and CO_2_

Pots of *O. alismoides* plants from the glasshouse were placed in white plastic buckets (25 × 25 × 35 cm) containing ~20 litres of tap water; two pots per bucket. Sixteen buckets were placed in a constant temperature room at 25 ± 2 °C and were illuminated with FSL T5/865 28 W fluorescence tubes on a 14 h light (08.00–22.00 h), 10 h dark photoperiod. The tap water was renewed twice a week. The plants were grown at two CO_2_ concentrations. A low CO_2_ concentration was produced by the natural photosynthetic activity of the plants which depleted the inorganic carbon concentration of the water, and increased the pH (from 8.32 to >9.0), with a CO_2_ concentration range of ~0.3–85 µmol l^−1^ and a mean of 11 µmol l^−1^. The high CO_2_ concentration was produced by adding CO_2_-saturated tap water to the buckets twice each day to reduce the pH to between 6.8 and 7.0, which generated on average 405 µmol l^−1^. The corresponding CO_2_ concentration over the whole growth period was between 136 µmol l^−1^ and 455 µmol l^−1^, with a mean of 286 µmol l^−1^. The buckets were gently stirred to mix the water before measurement and after each addition of CO_2_ solution. The buckets were covered with neutral-density shading material that produced a low level of light 25–30 µmol photon m^−2^ s^−1^, photosynthetically active radiation [PAR; Li-Cor underwater sensor (UWQ) connected to a Li-Cor LI-1400 data logger]. Alkalinity was measured every 2 d, and pH was measured every day (see methods below). Four days before the measurements, half the plants at high and low CO_2_ were exposed to a moderately high light at 150–165 μmol photon m^−2^ s^−1^ by removing the neutral-density shading material. There were therefore four treatments: high light and high CO_2_ (HLHC), high light and low CO_2_ (HLLC), low light and high CO_2_ (LLHC), and low light and low CO_2_ (LLLC), with the CO_2_ treatment lasting for 18 d and the high light treatment lasting for 4 d.

Whole leaves were collected at 07.30 h (just before the start of the photoperiod), 14.00 h, and 21.00 h (at the end of the photoperiod). Leaves grown at low concentrations of CO_2_ tended to have a layer of marl on the upper surface which was gently removed by rubbing. Leaves were immediately placed on aluminium foil on top of ice in a polystyrene box and kept in the dark to reduce metabolic changes. Three leaves per treatment were collected on each occasion from different plants in two or three different tanks. Each whole leaf was photographed, blotted gently with paper towels, and quickly weighed to determine the fresh weight. Each leaf was then cut in half down the mid-rib; one half was used for the determination of starch and photosynthesis enzymes, the other half for the determination of acidity. Each part of the leaf was photographed, placed in a pre-weighed foil envelope, weighed, and placed on ice. The procedure for processing 48 leaves took ~50 min. The foil envelopes containing the samples were stored in liquid nitrogen for later determination of acidity, starch, and enzyme activities.

### Response to short-term exposure to high CO_2_

Sixteen plants from the greenhouse, grown at high light and low CO_2_, were transferred in the early evening to the buckets and placed in the constant temperature room in tap water at high light (180 µmol photon m^−2^ s^−1^). Of the eight buckets, CO_2_-saturated tap water was added to four to produce high concentrations of CO_2_ (HC) as described above, while four tanks were untreated to retain a low concentration of CO_2_ (LC). The next day, whole leaves were collected just before 08.00 h (the start of the photoperiod), 10.00, 12.00, 14.00, 17.00, and 20.00 h (1 h before the end of the photoperiod). Three leaves per treatment were kept on ice and treated as above, except that a piece of the leaf was also used to measure rates of oxygen exchange. Chlorophyll content and fluorescence yield were also measured as described below.

### Response to short-term exposure to light and to different CO_2_ concentrations at night

Plants of *O. alismoides*, collected from the glasshouse at high light and low carbon, were placed in the growth room overnight and then exposed during the day to a photon irradiance of 150 µmol photon m^−2^ s^−1^ (HL treatment) and 15 µmol photon m^−2^ s^−1^ (LL treatment), both at low carbon. Leaves were collected as before in triplicate at 08.00, 12.00, 16.00, and 20.00 h, and stored in liquid nitrogen. During the subsequent night, the remaining plants from the two light treatments were incubated at two different CO_2_ concentrations (low or high CO_2_, produced as before) in the dark for 10 h. Three leaves per treatment were harvested at 07.30 h, before the onset of light, and stored in liquid nitrogen. Leaves were analysed for acidity, starch, malic acid, and enzyme activities.

### Measurement of acidity

Acidity was detected as described previously (Zhang *et al.*, 2014). Briefly, 10 ml of CO_2_-free milliQ water was added to a known fresh weight of leaves (0.2–0.5 g) that had been stored in liquid nitrogen and then boiled for 30 min. Acidity was measured by titration of the sample with 0.01 N NaOH to an endpoint of pH 8.3.

### Measurement of malic acid

A known fresh weight of frozen leaves (~0.4 g) was homogenized in 3 ml of ice-cold 0.6 N perchloric acid. The extract was centrifuged at 12 000 *g* for 10 min at 4 °C. The supernatant was neutralized with 5 mol l^−1^ K_2_CO_3_, centrifuged again, and assayed for malic acid following Delhaize *et al.* (1993).

### Measurement of starch

A known fresh weight of frozen leaves (~0.5 g) was homogenized in 5 ml of 80% ethanol. The homogenate was boiled for 3 min and centrifuged at 6000 *g* for 10 min at room temperature. The pellet was washed with 80% ethanol and this was repeated until the solution was colourless. The pellet was boiled for 10 min and, after cooling, 0.5 ml of 0.2 M Na acetate (pH 5.5) was added and the starch concentration was determined by the amyloglucosidase assay (Smith and Zeeman, 2006).

### Measurement of enzyme activities

The extraction and assay of PEPC, Rubisco, and pyruvate phosphate dikinase (PPDK) were based on the methods described previously (Zhang *et al.*, 2014). All these enzyme activities were assessed from the rate of appearance or disappearance of NADH at 340 nm at 25 °C measured using a microplate reader (Tecan M200 PRO, Austria).

### Measurement of chlorophyll, dry weight, and leaf area

About 0.1 g FW of leaf was extracted in 5 ml or 10 ml of ethanol and left overnight at 4 °C or boiled until the leaves were colourless, before measuring absorbance with a spectrophotometer at 649, 665, and 750 nm. Concentrations of Chl *a* and *b* were calculated using equations in Brain and Solomon (2007). Dry weight was measured after the leaves from HLLC in the glasshouse were dried for 48 h at 80 °C. Projected (one-sided) leaf area was calculated from digital photographs using AreaAna software (Huazhong University of Sciences and Technology, China), using squares of known area as reference.

### Measurement of pH and alkalinity

The alkalinity in the solution was measured by Gran titration with a standard solution of HCl. pH was measured with a combination pH electrode (E-201F, Shanghai Electronics Science Instrument Co., China) connected to a Thermo Orion Dual Star Benchtop pH/ISE Meter.

### Measurement of fluorescence yield

Fluorescence was recorded on six replicate leaves with a chlorophyll fluorometer (PAM-win, Walz, Germany) with a leaf clip attachment. The maximal photochemical efficiency of PSII (*F*_v_/*F*_m_) was measured 10 min before the start of the photoperiod, and subsequently yield (the actual photochemical efficiency of PSII) was measured immediately after illumination and every 5 min thereafter for the first hour. *F*_v_/*F*_m_ and the yield were obtained using the Win Control (3.25) software.

### Oxygen exchange

Oxygen exchange was measured with an optical oxygen electrode (Unisense OX-13298 and a Unisense microsensor multimeter Version 2.01) in a glass and Perspex chamber with a volume of 62 ml. A magnetic stirring bar at the base of the chamber, activated by an external motor, produced a steady flow of water around the chamber. The chamber was placed in a constant temperature water bath at 25 °C and illuminated from the side by fluorescent tubes (36 W, 6500 K colour temperature) that produced a photon irradiance (PAR) of 115 μmol photon m^−2^ s^−1^. The oxygen electrode was calibrated in the chamber with tap water (alkalinity ~1.8 mequiv l^−1^) that had been vigorously bubbled with air from outside the laboratory (100% saturation) and in tap water that had been vigorously bubbled with nitrogen (0% saturation). Leaves with an average FW of ~0.4 g and a projected area of ~28 cm^2^ were rinsed in tap water and placed in the chamber with tap water that had been bubbled with outside air and then bubbled with nitrogen for a few seconds to reduce the oxygen concentration. The concentration of CO_2_ at air equilibrium was calculated to be 14 µmol l^−1^ for an assumed outside air CO_2_ of 400 ppm. Tap water that had been bubbled vigorously with pure CO_2_ at 25 °C was calculated to have a CO_2_ concentration of 34.13 mmol l^−1^. Small volumes of this solution were added to produce 100 µmol l^−1^ CO_2_ (0.16 ml), followed by 625 µmol l^−1^ CO_2_ (0.97 ml). Rates of oxygen change were recorded for 5–10 min for each CO_2_ concentration. At the end of the measurement of photosynthesis, the chamber was placed in the dark and the decline in oxygen concentration was followed for ~15 min. O_2_ concentrations were recorded on a laptop connected to the Unisense meter, and rates of photosynthesis were calculated by linear regression in Microsoft Excel.

### Statistical analysis

All data presented in this study are the average ±SD. The data were analysed using SPSS 16.0 (SPSS Inc., Chicago, IL, USA). Treatment and time significance were determined with two-way and one-way ANOVA, and means denoted by different letters were significantly different at *P*<0.05 based on Duncan’s and Tukey’s post-hoc tests.

## Results

### CO_2_ concentrations based on pH and alkalinity

The high and low carbon treatments produced very different CO_2_ concentrations. Over the whole set of experiments, the average CO_2_ concentration was 286 µmol l^−1^ in the HC and 11 µmol l^−1^ in the LC treatment. There was no consistent diurnal pattern at HC, but at LC in the frequent measurements involved in the short-term responses to CO_2_ (below) the concentration of CO_2_ was 3.1 µmol l^−1^ at the start of the photoperiod and 0.6 µmol l^−1^ at the end. The concentration of HCO_3_^−^ in this experiment was 1.81 mmol l^−1^ in the HC treatment and fell from 0.47 mmol l^−1^ to 0.39 mmol l^−1^ during the day in the LC treatment.

### Acclimation to light and CO_2_

**Fig. 1.**
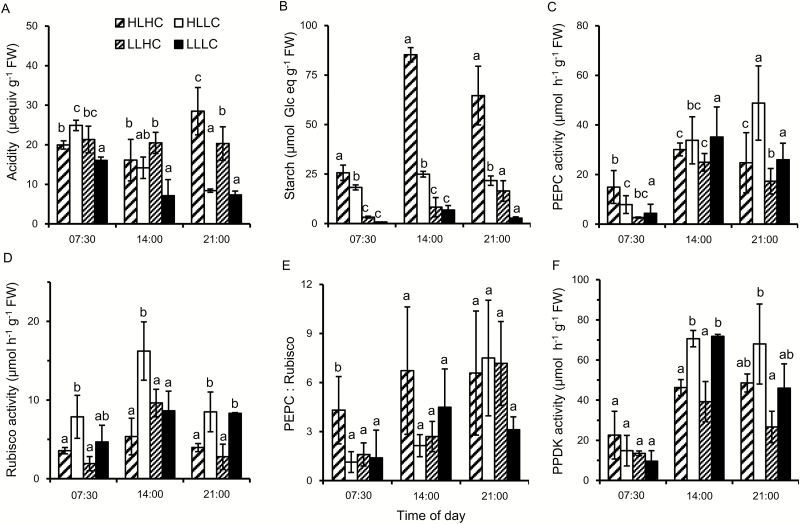
Diurnal changes in acidity, starch content, and enzyme activities in *O. alismoides* acclimated to a factorial combination of high light (HL), low light (LL), high CO_2_ (HC), and low CO_2_ (LC). (A) Acidity, (B) starch content as glucose equivalents, (C) PEPC activity, (D) Rubisco activity, (E) the PEPC:Rubisco ratio, and (F) PPDK activity. Bars show the mean with 1 SD for three replicates. Data with different letters are significantly different within a specific time (*P*<0.05; one-way ANOVA).

Biochemical and physiological properties of *O. alismoides* were compared after acclimation to low and high carbon (LC and HC) for 18 d and to low and high light (LL and HL) for the final 4 d. Night-time levels of acidity were similar across the treatments, varying between 17 µequiv g^−1^ FW and 25 µequiv g^−1^ FW (Fig. 1A). The average ratio of dry to fresh weight was 0.062 (SD is 0.007, *n*=25) so the acidity is equivalent to 1 µequiv g^−1^ DW and 1.55 µequiv g^−1^ DW. In the LC plants, there was a diel change in acidity of 17 µequiv g^−1^ FW at HL and 9 µequiv g^−1^ FW at LL, suggesting that CAM activity was present. In contrast, there was no diel change in acidity in the HC plants at HL or LL, because of a lack of decarboxylation, signifying the absence of CAM. High or low light did not affect the pattern of change, but the magnitude of change was lower in the LLLC plants than in the HLLC plants. An ANOVA consequently showed highly significant effects on acidity content of treatment and time of day, and there was an interaction between the two factors (see Supplementary Table S1 at *JXB* online). Starch content was lowest at the end of the night and increased during the day, and on average was statistically highest in the HLHC plants and statistically lowest in the LLLC plants (Fig. 1B; Supplementary Table S1). The activity of PEPC was lowest at the end of the night and highest during the day (Fig. 1C). Daytime PEPC activities were between 20 µmol g^−1^ FW h^−1^ and 50 µmol g^−1^ FW h^−1^, and similar across treatments (Supplemetnary Table S1). Rubisco activity tended to be higher in the middle than at the start and the end of the day (Fig. 1D).

Surprisingly, in plants acclimated to HLHC, the PEPC:Rubisco ratios were always >4 (Fig. 1E). In plants from the LC treatment, the PEPC:Rubisco ratio was 1 at the end of the night and increased to 6 by the end of the day for HL and to 3 for LL. In all the treatments, the activity of PPDK increased during the day, but the activity was lower in the HC than in the LC plants (Fig. 1F). There was a close relationship between the activity of PPDK and PEPC: on average, the activity of PPDK was 1.2 times greater than that of PEPC [linear regression: PPDK=1.23×(PEPC)+12.1; *R*^2^=0.62, *P*<0.001].

### Response to short-term exposure to high CO_2_

**Fig. 2.**
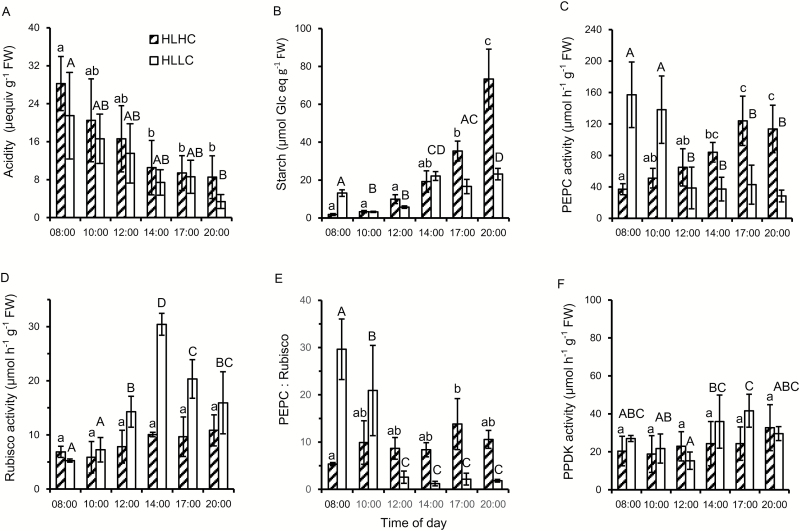
Effect of a short-term exposure to high CO_2_ on diurnal changes in acidity, starch content, and enzyme activities in *O. alismoides.* Plants acclimated to high light (HL) and low CO_2_ (LC) were exposed overnight to high CO_2_ (HC) and then kept at HLHC during the day. (A) Acidity, (B) starch content as glucose equivalents, (C) PEPC activity, (D) Rubisco activity, (E) the PEPC:Rubisco ratio, and (F) PPDK activity. Bars show the mean with 1 SD for three replicates. Data with different letters are significantly different within a specific treatment (*P*<0.05; one-way ANOVA).

Plants that were acclimated to high light and low CO_2_ were then exposed overnight to high and low CO_2_ at high light giving two treatments, HLLC and HLHC. The diel change in acidity was ~20 µequiv g^−1^ FW h^−1^ in plants kept either at LC or at HC (Fig. 2A). In both treatments, decarboxylation began immediately at the start of the photoperiod at a rate of 2–3 µequiv g^−1^ FW h^−1^, and was largely complete by 14.00 h. These results suggest that decarboxylation of the organic acid is not abolished by a high concentration of external CO_2_. Plants from the two treatments had similar starch contents until 14.00 h, which coincided with the time when the reduction in acidity had ceased (Fig. 2B). Subsequently, the starch content of plants in the HLLC treatment varied between 17 µmol and 23 µmol glucose equivalents g^−1^ FW, unlike that of plants in the HLHC treatment that increased markedly, up to 73 µmol glucose equivalents g^−1^ FW, by the end of the day. At high light, the activity of Rubisco was similar at HLLC and HLHC at the start of the day, but at 14.00 h the activity of Rubisco from plants at HLLC was 3-fold higher than that from plants at HLHC. The pattern of Rubisco activity at HLLC and HLHC was similar to that observed in the acclimation experiment, with 3-fold higher activity at 14.00 h in plants treated at HLLC (Fig. 2D). The PEPC activity of plants at HLLC was very high at the end of the night, nearly 160 µmol g^−1^ FW h^−1^, and the PEPC:Rubisco ratio was nearly 30 (Fig. 2C, E). In the HLLC treatment, the activity of PEPC and the PEPC:Rubisco ratio decreased in the morning and from 12.00 h onwards. In plants acclimated to HLHC, the activity of PEPC was lower than in the HLLC plants at the start of the day but increased significantly during the day, and reached 120 µmol g^−1^ FW h^−1^ and exceeded the activity in the HLLC plants from 12.00 h onwards. PPDK activity did not change significantly with treatment or time of day for the HLHC treatment and did not change markedly for the HLLC treatment (Fig. 2F; Supplementary Table S2).

**Fig. 3.**
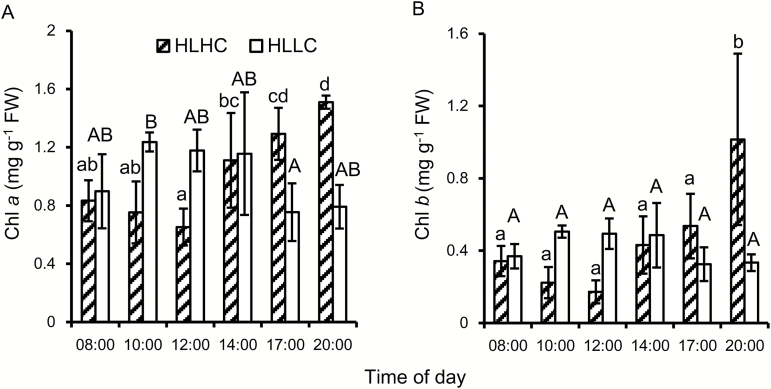
Effect of a short-term exposure to high CO_2_ on diurnal changes in chlorophyll content in *O. alismoides.* Plants acclimated to high light (HL) and low CO_2_ (LC) were exposed overnight to high CO_2_ (HC) and then kept at HLHC during the day. (A) Chl *a*, (B) Chl *b*. Bars show the mean with 1 SD for three replicates. Data with different letters are significantly different within a specific treatment (*P*<0.05; one-way ANOVA).

The contents of Chl *a* and Chl *b* (Fig. 3) were not significantly different for HLLC and HLHC during the day (Supplementary Table S2) but for HLHC they increased at the end of the day. Chl *a* content was always >2-fold higher than that of Chl *b*.

### Photosynthesis

**Fig. 4.**
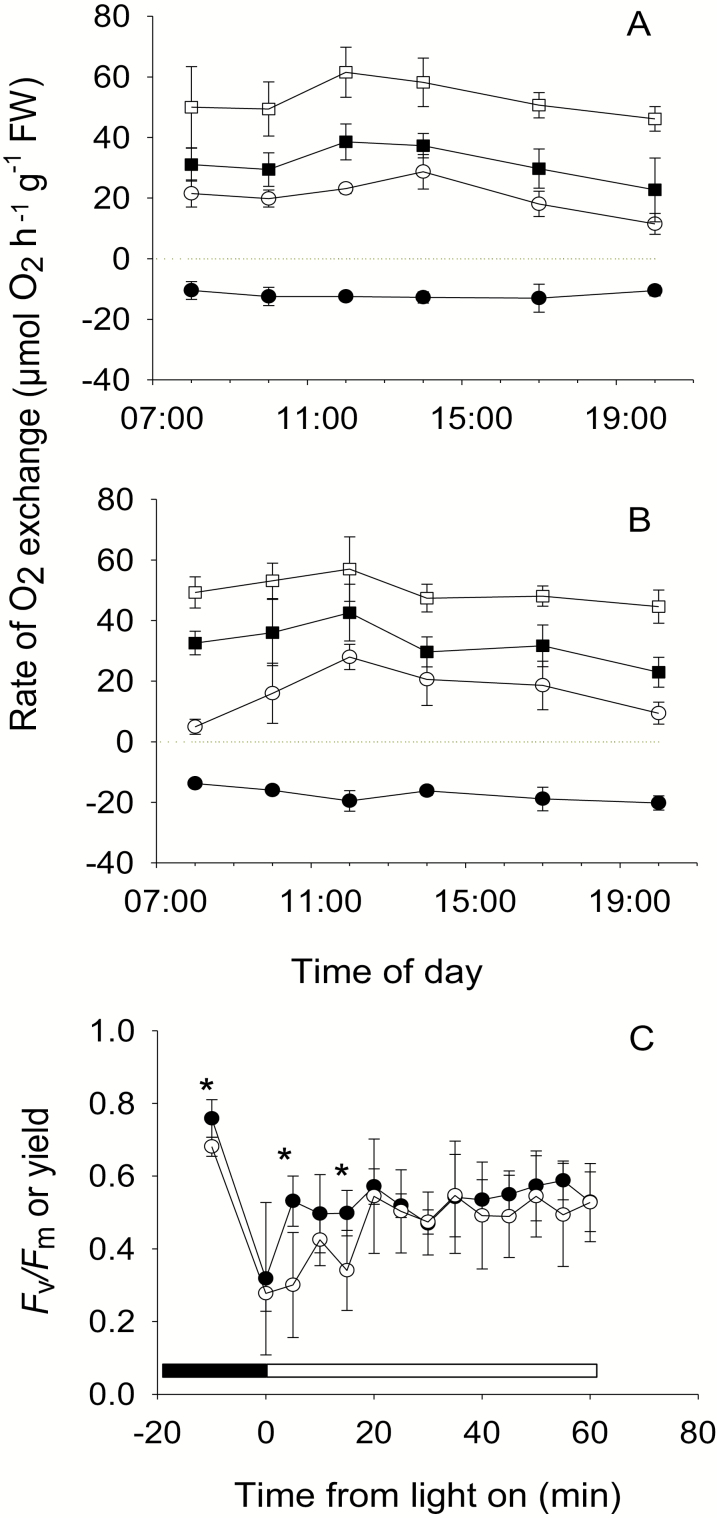
Diurnal changes in photosynthesis during the day and fluorescence at the start of the photoperiod in *O. alismoides* acclimated to high light and low CO_2_ (HLLC) and after a short exposure to high CO_2_ (HLHC). (A) Rate of net photosynthesis at 14 (open circles), 100 (filled squares), and 625 µmol l^−1^ CO_2_ (open squares) and respiration (filled circles) for HLHC leaves; (B) as for (A) but for HLLC leaves; (C) maximal and actual photochemical efficiency of PSII (*F*_v_/*F*_m_ and yield). Values are the mean with 1 SD for three replicates (A and B) or six replicates (C). In (C), a significant difference between treatments is indicated by an asterisk (*t*-test), and the dark and light periods are shown by horizontal bars.

Rates of O_2_ exchange for leaves from HLHC and HLLC treatments had different diurnal patterns. Rates at CO_2_ concentrations that were at, or close to, saturating changed little over the day for the HLLC treatment (Fig. 4B), but declined slightly after mid-day in the HLHC treatment as the starch content increased (Figs 2B, 4A). The rate of photosynthesis for HLHC at 14 µmol l^−1^ CO_2_ varied between 18 µmol O_2_ h^−1^ g^−1^ FW and 29 µmol O_2_ h^−1^ g^−1^ FW over the day, apart from at 20.00 h when it declined in line with the decline in the rate at saturating CO_2_. In contrast, the rate of photosynthesis for HLLC, although it followed a similar pattern after mid-day, was much lower at the start of the day and increased during the period when internal CO_2_ was being produced, reaching maximal rates at 12.00 h, and then declined. Rates of dark respiration measured during the day were, on average, 12 µmol O_2_ h^−1^ g^−1^ FW for HLHC leaves and did not vary markedly during the day. In contrast, dark respiration rates were higher for HLLC leaves, on average 17 µmol O_2_ h^−1^ g^−1^ FW, and they tended to increase during the day in absolute values and significantly as a proportion of rates at saturating CO_2_ (linear regression; *R*^2^=0.97, *P*<0.001).

Chlorophyll fluorescence analysis showed that *F*_v_/*F*_m_ was high at night and the yield decreased markedly at the onset of light (*t*-test, *t*=3.29, *P*<0.01). After this decrease, at HLHC, PSII yield increased rapidly to a steady-state value of ~0.54, within ~20 min. However, at HLLC, the rate of recovery was lower (Fig. 4C) and there were significant differences between HLLC and HLHC at 08.05 h and 08.15 h (*t*-test, *t*=3.52, *P*<0.01 and *t*=3.05, *P*<0.05, respectively).

### Response to short-term exposure to light and to different CO_2_ concentrations at night

**Fig. 5.**
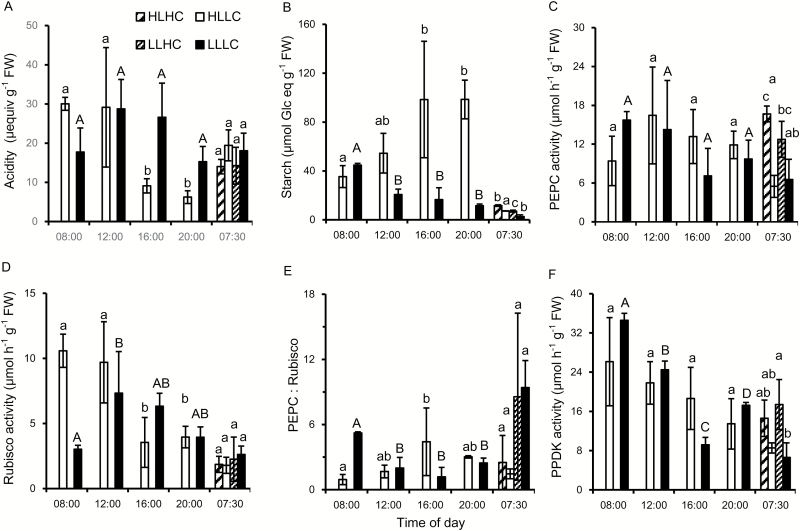
Effect of a short-term exposure to low light on diurnal changes in acidity, starch content, and enzyme activities in *O. alismoides.* Plants acclimated to high light (HL) and low CO_2_ (LC) were kept at low CO_2_ and exposed to low light (LLLC). At the end of the photoperiod, plants were treated overnight at high CO_2_ (HC) or kept at low CO_2_, and leaves were harvested at the end of the scotoperiod. (A) Acidity, (B) starch content as glucose equivalents, (C) PEPC activity, (D) Rubisco activity, (E) the PEPC:Rubisco ratio, and (F) PPDK activity. Bars show the mean with 1 SD for three replicates. Data with different letters are significantly different within a specific treatment. At the end of the scotoperiod (07.30 h), different letters designate treatments whose results are significantly different (*P*<0.05; one-way ANOVA).

When plants acclimated to HLLC were transferred to LLLC, the acidity content during the day did not change significantly, whereas it decreased significantly in the HLLC plants (Fig. 5A).These plants also displayed very different patterns of starch content during the day (Fig. 5B). In plants at HLLC, the starch content increased significantly, while in plants at LLLC, the starch content decreased monotonically to 11.5 µmol glucose equivalents g^−1^ FW by the end of the day. At the end of the next night, the starch content had decreased to 7.2 and 2.6 µmol glucose equivalents g^−1^ FW for HLLC and LLLC treatments, respectively, but plants supplemented with CO_2_ overnight had between 4.4 and 4.5 µmol glucose equivalents g^−1^ FW more starch than plants treated with low CO_2_ overnight.

There were no significant effects of the short-term light treatment on the activity of PEPC during the day (Fig. 5C). Rubisco activity was higher during the middle of the day for the LLLC treatment and declined during the day for the HLLC treatment (Fig. 5D). The PEPC:Rubisco ratio was highest at the start of the day for the LLLC treatment and increased during the day in the HLLC treatment (Fig. 5E).The PEPC activity in plants exposed overnight to high CO_2_ was greater at the end of the night than that in plants at low CO_2_. There was no significant effect of the light treatment on the activity of PPDK, and activity did not decrease significantly at HL but decreased significantly at LL during the day (Fig. 5F; Supplementary Table S3).

### Contribution of CAM

Generally there was an inverse relationship between acidity and starch content. The expected linear relationship was found in the morning between malate expressed as 2H^+^ equivalents, and acidity (malate=1.086×acidity+4.14, *R*^2^=0.83). We assumed that 1 equivalent of acidity represents 0.5 mol of malate, that for every mole of malate (C4) decarboxylated 1 mol of carbon (C+C_3_) is produced, that every mole of glucose (C6) equivalent represents 6 mol of carbon, and therefore that every acidity equivalent removed represents a 12th of a mole of glucose. Assuming also that no other processes are involved in changes in starch and acidity apart from non-CAM photosynthesis, diurnal changes in acidity and starch were used to estimate the contribution of malate decarboxylation to starch formation. On this basis, at low carbon, malate decarboxylation contributes up to 21% of the carbon stored in starch, but <2.5% for plants at high carbon (Table 1).

**Table 1.** Contribution of diel acidity change to starch production in *O. alismoides* leaves during the day

Experiment	Treatment	Mean diel change in acidity (µequiv g^−1^ FW)	Percentage contribution of acid change to starch
Acclimation	HLHC	1.0 (3.1)	0.0
	HLLC	–8.5 (3.5)	20.7
	LLHC	8.8 (0.7)	0.6
	LLLC	16.5 (0.8)	12.1
Response to CO_2_	HLHC	19.7 (4.2)	2.3
	HLLC	18.1 (5.4)	7.6
Response to light	HLLC	23.8 (1.3)	2.2
	LLLC	2.5 (4.2)	0.5

Data represent the mean with the SD in parentheses.

The percentage of night-time respiration of carbon that could be conserved by the overnight accumulation of acid was calculated as in Klavsen and Maberly (2010). This was 1.5% for HLHC and 6% for HLLC plants. Both are lower than the equivalent values for *C. helmsii* of 11% and 32%, respectively.

## Discussion

### Three CCMs in *O. alismoides*

*Ottelia alismoides* has been shown to use bicarbonate and to have C_4_ metabolism, both constitutively, and to possess CAM facultatively, being induced by low CO_2_ (Zhang *et al.*, 2014; Yin *et al.*, 2017). The results presented here confirmed that C_4_ is present regardless of the CO_2_ concentration or light level. At HLLC, PEPC activity declined during the day but the PEPC:Rubisco ratio was between 1.2 and 2.6, in agreement with a previous report and in a range typical of terrestrial C_4_ plants (Zhang *et al.*, 2014). Moreover, the activity of other enzymes required for the operation of the C_4_ cycle, such as PPDK that regenerates PEP to ensure the supply of substrate for PEPC, was also high and greater at low versus high CO_2_.

In CAM plants, a high PEPC activity at night allows malic acid to be produced in the dark. In *O. alismoides*, the activity of PEPC in LC plants was very high at dawn, and the PEPC:Rubisco ratio was ~30, consistent with nocturnal carboxylation as a consequence of CAM activity. PEPC activity in plants exposed to HC at night was greater than in plants maintained at LC, consistent with greater CAM activity exploiting the nocturnal carbon reserves. The diel fluctuations in acidity found here, between 17 µequiv g^−1^ FW and 25 µequiv g^−1^ FW for plants at HLLC in the acclimation and short-term experiments, respectively, were slightly lower than those (up to 34 µequiv g^−1^ FW) found in Zhang *et al.* (2014). They are also lower than those reported in the literature for some species (Keeley, 1981, 1998*b*) but higher or similar to those reported in a number of other putative CAM species (Webb *et al.*, 1988; Keeley, 1998*b*; Yin *et al.*, 2017). Overall, these enzyme activities and their patterns of change confirm that *O. alismoides* grown at LC can operate CAM during the night and C_4_-like metabolism during the day.

### Regulation of CAM

In aquatic CAM species, such as *Littorella uniflora* and *Isoetes kirkii*, both light and CO_2_ affect CAM activity (Madsen, 1987; Robe and Griffiths, 1990; Hostrup and Wiegleb, 1991; Rattray *et al.*, 1992). Although *O. alismoides* was apparently co-limited by light and inorganic carbon when grown at low light and low CO_2_, CO_2_ seems to be a very effective regulator of CAM, because CAM was absent at high CO_2_. The importance of CO_2_ in controlling and inducing changes in CAM activity in *O. alismoides* is consistent with the hypothesis of Keeley (1981) suggesting that CAM has been selected as a mechanism for enhancing net carbon gain in inorganic carbon-limited aquatic environments. Investment in CAM enzymatic apparatus and energy is beneficial when the inorganic carbon supply is limiting but not when other environmental variables, such as light or nutrients, are limiting (Madsen, 1987; Baattrup-Pedersen and Madsen, 1999). In *O. alismoides*, CAM was down-regulated when grown at ~25 µmol photon m^−2^ s^−1^ compared with ~150 µmol photon m^−2^ s^−1^, similarly to *L. uniflora* (Madsen, 1987; Baattrup-Pedersen and Madsen, 1999) and *C. helmsii* (Klavsen and Maberly, 2010). Similarly, CAM in terrestrial plants is down-regulated when grown at low light (Borland *et al.*, 1996; Taybi *et al.*, 2002).

In aquatic CAM plants, a high concentration of CO_2_ during the day reduces the rate of decarboxylation, and therefore the acidity at the beginning and end of the day are similar (Keeley, 1982; Rattray *et al.*, 1992). For *C. helmsii*, the rates of decarboxylation are similar at different concentrations of CO_2_ (Klavsen and Maberly, 2010), and this is also the case in *O. alismoides* where it was 2–3 µequiv g^−1^ FW h^−1^ (equal to 32–48 µequiv g^−1^ DW h^−1^), but lower than that of *C. helmsii* (Klavsen and Maberly, 2010). In *C. helmsii*, the start of decarboxylation varied with CO_2_ (Klavsen and Maberly, 2010). In contrast, in *O. alismoides*, decarboxylation began immediately at the start of the day, even at high CO_2_, suggesting that decarboxylation of malic acid was unaffected by high CO_2_. Therefore, in *O. alismoides* and *C. helmsii*, the decarboxylation of malic acid is not influenced by CO_2_ but the effect of CO_2_ on timing during the light period differs between the two species.

Light intensity affects the decarboxylation of malic acid in *O. alismoides* as expected from other CAM studies (Osmond, 1978). When *O. alismoides* grown at HLLC were transferred to LLLC, the acidity content did not change at the start of the photoperiod, suggesting that decarboxylation of acid is not under circadian control but only occurs if sufficient light energy is available, as a direct effect or an indirect effect on carbon demand. As starch degradation supplies PEP for malic acid synthesis, one might expect an inverse relationship between starch accumulation and malic acid concentration, and therefore acidity. This is indeed a key characteristic in CAM plants (Borland and Taybi, 2004). In *O. alismoides*, the content of starch showed an inverse diel relationship to titratable acidity. When plants from HLLC were transferred to LL for a short period of time, the acidity content was high and did not change during the day, and the diel cycle of starch was abolished as in another CAM plant, *Aechmea ‘Maya’* (Ceusters *et al.*, 2008).

The contribution of acidity change to starch production in *O. alismoides* was estimated from concomitant changes in these variables. When CAM was active, it contributed up to 21% of the starch produced. The contribution from CAM to the carbon balance in *O. alismoides* is similar to that in *C. helmsii* (Klavsen and Maberly, 2010) but is less than in *Isoetes* and *L. uniflora* (Robe and Griffiths, 1990; Madsen *et al.*, 2002). In *O. alismoides*, CAM traps a relatively low proportion of night-time respiration but it does appear to have a beneficial effect on the rate of photosynthesis. At the start of the photoperiod, the rate of photosynthesis in plants at HLLC was low at air equilibrium CO_2_ concentrations but subsequently increased ~5-fold during the period of decarboxylation and internal production of CO_2_, and decreased again in the afternoon as the decarboxylation rate decreased. Furthermore, analysis of fluorescence kinetics confirmed that while photosynthesis by the plants was activated rapidly at HC, the plants at LC took ~20 min to reach their full activity, which is consistent with the activation of a CCM.

### Compatibility between C_4_, CAM activity, and bicarbonate use

Many aquatic macrophytes can supplement normal C_3_ photosynthesis by additional strategies for C gain such as CAM, C_4_ metabolism, or bicarbonate use (Klavsen *et al.*, 2011). *Ottelia alismoides*, perhaps uniquely, appears to have all three of the above-mentioned strategies (Zhang *et al.*, 2014). The compilation of macrophyte CCMs in Maberly and Madsen (2002) suggested that no CAM plants can use bicarbonate. In addition to *O. alismoides*, a possible additional exception is another species from the Hydrocharitaceae, *Vallisneria spinulosa*, which can use bicarbonate and perform low-level CAM (Yin *et al.*, 2017). The explanation for the rarity of this combination of CCMs is not known, but might relate to habitat. For example, CAM is common in isoetids which tend to grow at sites with low concentrations of bicarbonate and have exploitation strategies involving uptake of CO_2_ from the sediment via continuous lacunae between roots and leaves. The two elodeids *O. alismoides* and *V. spinulosa* do not have this option and typically are found at sites with high bicarbonate concentrations. More work is needed to understand why bicarbonate use and CAM is a rare combination of CCMs in aquatic plants. *Ottelia alismoides* is an annual aquatic plant and is invasive in several regions of the world (e.g. it is on the USA’s list of introduced, invasive, and noxious plants; https://plants.usda.gov/core/profile?symbol=OTAL, last accessed 21 February 2017). It generally inhabits still or slow-flowing water, and can produce a high plant biomass causing large diurnal fluctuations and low concentrations of dissolved CO_2_. The flexibility conferred by possessing three CCMs (bicarbonate and CO_2_ use, C_4_ and CAM metabolism) may help explain its ecological success and allow it to complete its growth cycle within a year.

CAM and C_4_ do not usually co-exist in terrestrial plants, despite the similarity of their biochemical processes. Some species in the genus *Portulaca* operate C_4_ photosynthesis but also CAM under drought conditions (Koch and Kennedy, 1982; Guralnick *et al.*, 2002). However, in the best studied species, *P. grandiflora*, CAM and C_4_ do not occur in the same cells (Guralnick *et al.*, 2002). Sage (2002) suggests that while low-level CAM can co-exist with C_4_ photosynthesis, albeit in different parts of the leaf based on the *Portulaca* example, there are a number of morphological and biochemical incompatibilities that prevent these two photosynthesis mechanisms from co-existing. One key difference between aquatic and terrestrial CAM plants is the role that stomata play in controlling carbon uptake from air. In water, if PEPC is not down-regulated during the night, and if biochemical intermediates are available, continued PEPC activity will lead to the accumulation of malic acid. *Ottelia alismoides* is currently the only known species where C_4_ and CAM appear to be present in the same tissue, given that its leaf is only two cells thick, although further studies are needed to establish the precise location of these two biochemical pathways, determine how futile cycling is prevented during C_4_ carbon fixation, and understand how the carboxylation and decarboxylation enzymes are regulated.

## Supplementary data

Supplementary data are available at *JXB* online.

Table S1. Results of ANOVA for physiological parameters in *O. alismoides* grown under four combinations of light and CO_2_, with treatment and time as factors.

Table S2. Results of ANOVA for physiological parameters in *O. alismoides* treated with short-term variable CO_2_ during the day, with treatment and time as factors.

Table S3. Results of ANOVA for physiological parameters in *O. alismoides* treated with short-term variable light at day and variable CO_2_ concentration at night, with treatment and time as factors.

## Supplementary Material

Supplementary_Tables_S1_S3
