# Supplementary material for: Responses of Ottelia alismoides, an aquatic plant with three CCMs, to variable CO2 and light
Source: J Exp Bot. 2017 Mar 21;68(14):3985–95. doi: 10.1093/jxb/erx064 (PMC5853927; doi:10.1093/jxb/erx064)
Supplement: Supplementary_Tables_S1_S3 [file erx064_suppl_supplementary_tables_s1_s3.pdf]

Table S1. *Results of ANOVA for physiological parameters in* Ottelia alismoides *grown under four combinations of light and CO_2: low light and low CO_2 (LLLC), low light and high CO_2 (LLHC), high light and low CO_2 (HLLC), high light and high CO_2 (HLHC),respectively, with treatment and time as factors.*

Variable	Treatment	Time	Interaction
Acidity	***	***	***
Starch	***	***	***
PEPC	**	***	*
Rubisco	***	***	*
PPDK	***	***	**

***P<0.001, ** P<0.01, * P<0.05.

Table S2. *Results of ANOVA for physiological parameters in* Ottelia alismoides *treated with short-term variable* CO_2 *during the day, with treatment and time as factors.*

Variable	Treatment	Time	Interaction
Acidity	NS	***	NS
Starch	***	***	***
PEPC	NS	*	
Rubisco	***	***	***
PPDK	NS	NS	NS
Chl *a*	NS	NS	***
Chl *b*	NS	NS	**
Total Chl	NS	NS	***

***P<0.001, ** P<0.01, * P<0.05, NS means no significant.

Table S3. *Results of ANOVA for physiological parameters in* Ottelia alismoides *treated with short-term variable light at day and variable CO_2 concentration at night, with treatment and time as factors.*

Variable	Treatment	Time	Interaction
Acidity	NS	**	*
Starch	***	NS	**
PEPC	NS	NS	NS
Rubisco	*	**	**
PPDK	NS	***	*
Malic acid	NS	***	**

***P<0.001, ** P<0.01, * P<0.05, NS means no significant.
